# On Physicians Extracting Teeth

**Published:** 1853-04

**Authors:** C. P. Culver

**Affiliations:** New Castle, Ky.


### *On Physicians Extracting Teeth.* By C. P. Culver, New-Castle, Ky., Jan. 3d, 1853.

I seek not to controvert a long established and time honored custom; but simply to suggest a few thoughts touching a question, which is of interest to the profession, especially that of dentistry.

I have long been of the opinion, that operations pertaining to the mouth, came more directly within the province of the dentist than the physician. It is one of the prime features of the dentist's profession, to perform surgical operations in the mouth, and to treat all diseases thereof, of whatever nature or character they may be, of which the human family may be afflicted. Therefore, their operations are not alone confined to the mechanical laboratory, supplying, artificially, the loss of the dental organs sustained by disease, the physician's "*Lion*" —or perchance by accident; but, with the present expansion of knowledge, and the advancement of science, the dentist skilled in his profession, (none others are worthy of the title of dentist,) is required to *preserve* the natural organs, which, from various causes, have become involved in disease, It is well known to the dental profession, as well as the medical, that there are many teeth, which have become involved in disease, that with proper treatment, could have been saved, to continue their office of masticating food, of decorating and beautifying the contour of the human face; but for this "time honored custom" of going to the physician instead of the dentist, as soon as these ornaments of nature have become the seat of pain; and the physician, without an inquiry as to the possibility, saying nothing of the propriety of saving the organ, fastens his "*key*" upon it, (for few have any other instrument) and out comes the offending member.

I am aware, that the medical profession claim priority to the practice of surgery, of whatever nature or character it might assume; but I do most emphatically question their knowledge, as well as their skill, in performing operations upon the teeth. It is the duty of the physician, to save life, and not to destroy it—to preserve the natural organs of the human economy, and not to divest man of any of these, unless, by sound judgment, based upon a proper knowledge of the character of the disease, and the peculiar characteristics of the members to be operated upon, it becomes the *dernier* resort—the last alternative. How different is this, with most of our medical practitioners, in relation to the teeth—I go farther, and say, that *no* surgical operation should ever be performed, unless by the judicious and skillful, *and with the proper instruments.*

With the increased knowledge in the profession of Dental Surgery, and the facilities offered by them, for performing surgical operations in the mouth, I would ask, first if it would not comport more with the *dignity* of the medical profession, to refer patients, desirous of having their teeth extracted, or of having any other diseases of the mouth treated, to the dentist, instead of attempting, themselves, to perform operations, which most of them, at best, are illy prepared to perform? I have seen, as well as others of experience and observation, badly performed operations within the mouth, by the use of the "key instrument," when in the hands of the unskillful, as well as direful consequences to the patient.

I have had numerous cases come under my own observation and treatment, of individuals, who, from the use of the "key," in the hands of those who seldom stop to inquire into the possibility of an existing state of anchyloses of the periostial membrane with the teeth and sockets, and consequently, in their extraction, large portions of the jaw-bone have been removed with the teeth—gums torn and mashed, leaving the necks of a portion of the remaining teeth exposed, which, in time, become the seat of disease, terminating in ulcers, necroses of the jaw-bone, and frequently, tumors of a a painful and distorted character, producing the most excruciating agonies to the patients, and ultimately death; all arising from a badly performed operation in the extraction of a single tooth.

With the little attention given to the study of the diseases of the teeth and gums, by the mass of our practicing physicians throughout the country, would it not, secondly, be more in conformity with the interest of both professions, or if you please, both branches of the same profession, for the physician to refer all operations of this character to the dentist, and the dentist in return, refer to the physician all his patients who require some general medical treatment, before local treatment for diseases of the mouth can be attended with any degree of success. In this way, both physician and dentist would be mutually benefitted—patients saved a vast deal of pain, and in the end, an amount of money.

The amount which the physician makes annually, in a section of country, or city, where there is a dentist, by extracting teeth at *twenty-five cents* each, or even at fifty, would not be equal to the amount of business which the dentist could place within his hands, where he was doing a reasonable practice.

There are comparatively few patients who call upon me, desiring treatment for diseases of the gums or palitine organs, that I do not, in the first place, prescribe some general treatment; which, if there were more *unanimity* of feeling in the professions, I would refer to the physician, rather than to administer myself—I am far from implying however, that the dentist should not possess a knowledge of the necessary treatment to be adopted, or the physician the nature and character of all diseases pertaining to the dental organs; but upon the contrary, that each should understand the general nature and character if you please, of all diseases, and their treatment:—though not intimating from this fact, that it necessarrily follows, in virtue of this knowledge, that he should practise indiscriminately, when occasion presents itself, dentistry and medicine. Notwithstanding the professions may be viewed as indistinct in their fundamental elements, yet as they are separated as branches of the same profession, then, and are therefore carried to greater perfection by those giving their whole attention to one or the other; and the firm or elevated stand amid a multitude of almost insurmountable obstacles, which the dental profession have taken to elevate the branch of the physical sciences, by affording by their skill and undaunted perseverance, increased facilities for performing surgical operations upon the mouth, as well as an increased amount of information pertaining to diseases thereof, besides lessening very considerably, the amount of suffering to patients; and these facts being acceeded to by the medical branch of the profession, should ever secure to the dentist, skilled in his profession, the confidence and support of the physician.

The idea of a dental chair in our medical colleges, has originated, and is now being advocated, from a knowledge of this want of efficient operators in the medical profession generally, when it comes to operations upon the teeth; and the many *bungling* operations performed by the medical practitioner, to the discredit of the profession, and the lasting discomfiture of the patient.

Were the physician, in my estimation, to attempt to qualify himself properly for the practice of medicine and dental surgery, at the same time, and then attempt to apply them in practise as occasion might require, he would find himself "swamped" many times, and unable to extricate himself, during which, one branch or the other of his profession would inevitably suffer. But, as men are inclined to pursue such branches of the various avocations of life, as are best adapted to their nature and capacities, and more especially so with those who adopt the professions, to follow such branches of them, as are best adapted to their taste, and in which they can excel, and by devoting their attention to, may carry to greater perfection. Hence, the necessity of forever keeping these two branches, of the same science, if you please to consider them, separate and distinct in their practise.

The man who confines himself alone to the study and practise of Surgery, is infinitely better qualified to perform surgical operations. The physician, who gives his time and attention to the study and treatment of fevers, and other diseases of the human constitution, is better qualified to do justice to his patients than the surgeon, who has comparatively no taste for the practice of medicine. So with the Oculist in the treatment of diseases of the eye. The Dentist in the treatment of diseases of the teeth, &c., &c.

I might say more to advantage, but will content myself with suggesting to the medical profession the importance of considering this matter in its proper light, and if the suggestions meet their approbation,—(and we cannot see why they should not,) and when they are aware of a skilful dentist being within reach, to refer their applicants, for extracting of teeth and other dental operations to him; and when such are claiming the patronage of the suffering public, to lay aside their "*rusty and aged old key*, and give him rest and repose.

